# Risk Assessment of Impairment of Fertility Due to Exposure to Tobacco Constituents Classified as Reprotoxicants

**DOI:** 10.3390/toxics13040234

**Published:** 2025-03-23

**Authors:** Carmen Estevan, Gabriela A. Báez-Barroso, Eugenio Vilanova, Miguel A. Sogorb

**Affiliations:** 1 Departamento de Biología Aplicada, Universidad Miguel Hernández de Elche, Avenida de la Universidad s/n, 03202 Elche, Spain; cestevan@umh.es; 2 Instituto de Bioingeniería, Universidad Miguel Hernández de Elche, Avenida de la Universidad s/n, 03202 Elche, Spain; alejandra.baez.barroso@gmail.com (G.A.B.-B.);

**Keywords:** risk assessment, infertility, tobacco, smoking, derived no effect level (DNEL), NOAEL, LOAEL, threshold mechanism

## Abstract

Background: Epidemiological studies demonstrate that exposure to tobacco causes infertility. A reference cigarette contains up to 47 chemicals above the quantification level, of which acrylamide, benzopyrene, cadmium, ethylene oxide and lead are classified as known (category 1A), presumed (category 1B) or suspected (category 2) human reproductive toxicants due to their effects on fertility and sexual function. Methods: We collected toxicological information on these substances to establish their respective systemic-derived no-effect levels (internal doses predicted not to alter fertility). We also estimated the systemic exposure to these four substances by smokers consuming 20 cigarettes per day. Results: The risks (ratios between exposure and safe dose) were 0.23, 0.06, 0.18, 0.01 and 0.00002 for acrylamide, benzopyrene, cadmium, ethylene oxide and lead, respectively. The combined risk was 0.48. Conclusions: It was concluded that the changes in fertility resulting from the consumption of the substances in tobacco classified as toxic to fertility could not be explained by mechanisms with a toxicity threshold attributable to these five substances. No safe dose could be derived for tobacco use in persons seeking pregnancy; this applied to both active and passive smokers.

## 1. Introduction

The World Health Organization (WHO) recognizes tobacco use as one of the greatest threats to public health due to its widespread use worldwide [1]. According to 2020 data, 22.3% of the world’s population uses tobacco [1]. This figure is similar in Europe where, according to EUROSTAT, 19% of the population was recognized as daily smoker in 2020 [2].

Reproductive toxicity, or reprotoxicity, is a term that encompasses adverse effects on the reproductive system. It includes changes not only in fertility but also in embryo or fetal development caused by exposure to xenobiotics [3]. Tobacco use is one of the environmental factors that can cause reproductive changes in both fertility and development. Developmental toxicity is a specific type of reproductive toxicity referring to the adverse effects on the growing organism from the embryonal state to the time of an individual’s sexual maturation [3].

The World Health Organization (WHO) recognizes infertility as a condition that affects both men and women and occurs when conception fails despite prolonged attempts [4]. Epidemiological studies suggest that lifestyle factors, particularly smoking, may negatively affect fertility, as smoking is associated with reduced sperm quality in men and lower success rates with fertility treatments in women.

To provide a scientific basis for risk management, the aim of this work is to determine whether the fertility impairments observed in epidemiologic studies are due to mechanisms with or without a threshold of toxicity by compounds already classified as toxic to fertility. We performed a risk assessment of fertility toxicity derived from tobacco exposure. For this purpose, we compiled the available toxicological information on the fertility impairments of individual substances quantified and contained in a reference cigarette as defined by the Center for Tobacco Reference Products (CTRP) of the University of Kentucky (USA). We then used methods recognized by official international agencies for risk assessment to estimate a systemic (internal) derived no-effect level (DNEL) for effects with mechanisms with a toxicity threshold. Finally, we compared the exposure derived from smoking 20 cigarettes per day with such DNELs for individual substances and for the resulting mixture.

## 2. Materials and Methods

### 2.1. Cigarette Composition

The cigarette composition was obtained from the CTRP. The CTRP is part of the College of Agriculture, Food and Environment at the University of Kentucky and has provided standard products for non-clinical tobacco research since 1968. The current standard is called 1R6F [5]. To work with the worst reasonable case, we took the amount of each substance obtained from an intense smoking regime with a puff volume of 55 mL and a puff frequency of one every 30 s [6].

### 2.2. Classification and Labelling of Substances in Tobacco Cigarettes

The official classification and labelling of the chemicals contained in the reference cigarette 1R6F was obtained from the European Chemical Agency (ECHA) in accordance with the Classification and Labelling Regulation [7] and the Labelling Inventory of Notified and Registered Substances [8].

### 2.3. Reproductive Toxicity Studies

Information on the toxicity to fertility of the substances in tobacco classified as toxic to reproduction was obtained from the databases of worldwide regulatory agencies with specialised scientific committees that endorse the robustness of the information used. Specifically, the following agencies’ databases were used: (i) United States Environmental Protection Agency (USEPA); (ii) WHO (Environmental Health Criteria monographs); (iii) ECHA (Register of Chemical Substances and Mixtures (REACH)); and (iv) National Toxicology Program (NTP) monographs.

For those cigarette components that are not classified as toxic for fertility, additional information on their reproductive toxicity has been retrieved from the ECHA Register of Chemical Substances and Mixtures and from National Toxicology Program (NTP) monographs. This information is available in Supplementary Table S1.

### 2.4. Risk Assessment

The assessment of the risk to fertility associated with tobacco exposure was performed according to the four classical steps of the toxic risk assessment process for exposure to chemical substances: (i) hazard identification; (ii) hazard assessment; (iii) exposure assessment; (iv) risk characterisation.

#### 2.4.1. Hazard Identification

The hazard of the substances classified as toxic for reproduction has been identified as described in Section 2.2.

#### 2.4.2. Hazard Assessment

The objective of the hazard assessment was to establish an (internal) systemic DNEL for impaired fertility. The systemic DNEL is defined as the internal dose of the tested substance at which no effects on fertility are expected and was estimated according to the procedures used by ECHA for this purpose. The points of departure were the no observed adverse effect level (NOAEL) and the lowest observed adverse effect level (LOAEL) found in the toxicological information used to identify the hazard of each substance, which was obtained as described in Section 2.3. A DNEL was estimated for each NOAEL and LOAEL identified. To be as conservative as possible, the lowest systemic DNEL was chosen for the risk assessment. The DNEL was estimated in each case using the following formula:

$$Systemic\;DNEL=\frac{NOAEL\;\left(or\;LOAEL\right)\times Absorption}{Uncertainty\;factor\;(UF)}$$

The absorption factor was obtained from bibliographic searches as the percentage of the external dose that is capable of reaching the interior of the organism. That is, in oral exposure studies, the percentage of the substance ingested in the diet that is absorbed in the gastrointestinal tract, and in inhalation studies, the percentage of the substance inhaled by the animal that is absorbed in the lungs and distributed in the body via the blood.

The aim of a risk assessment is to protect human health from the adverse effects of exposure to chemicals. Therefore, if the studies used to determine the DNEL are animal studies, it is necessary to use uncertainty factors (UFs) to compensate for potential differences between humans and animals and to protect the most susceptible individuals. To estimate the DNEL, the default UFs set by ECHA in its guidance [9] were used and are presented in Table 1 and Table 2.

#### 2.4.3. Exposure Assessment

The aim was to determine the systemic (internal) exposure of tobacco smokers to the substances classified as toxic to fertility and sexual function. The concentration of each substance in the reference cigarette 1R6F was used. It was assumed that each smoker consumed 20 cigarettes per day and had a body weight of 70 kg. The inhalation absorption values found in the bibliography using the same databases searched to determine the hazard of each substance were taken into account. Exposure was thus estimated using the following formula:

$$Exposure=\frac{Concentration\times 20\times Absorption}{70}$$

#### 2.4.4. Risk Characterisation

Finally, the risk for the individual substances was estimated as follows:

$$Risk=\frac{Systemic\;Exposure}{Systemic\;DNEL}$$

Thus, if the ratio between the systemic exposure and the systemic DNEL (safe use based on reproductive effects) is greater than 1, it means that the exposure is higher than the DNEL (safe dose for reproductive effects) exposure and therefore risk of impairment of fertility due to exposure to the assessed substance is expected. Conversely, a risk ratio lower than 1 indicates that the exposure is lower than the safe dose and that there is no expected risk of alterations in fertility as a result of the exposure to the assessed substance.

Furthermore, the risk of the mixture is defined as the sum of the individual risk ratios for all the substances in the mixture:

$$Risk\;of\;the\;mixture=\sum i\left(\frac{Systemic\;Exposure}{Systemic\;DNEL}\right)i$$

## 3. Results

### 3.1. Hazard Identification of the Substances Contained in Reference Cigarette 1R6F

Reference cigarette smoke 1R6F contains 47 different quantified substances, some with harmonized classification and labelling according to the inventory database of ECHA [8] (Table 3). Of the substances quantified in the reference cigarette 1R6F, some are classified as mutagenic, carcinogenic, irritant and corrosive to the skin and the eyes, skin sensitizers, toxic to various organs after one or several exposures, acute toxicity by any of the three routes and toxic for reproduction (Table 3). Five different substances are classified as toxic for fertility and sexual function: acrylamide, benzo[a]pyrene, cadmium, ethylene oxide and lead (the grey entries in Table 3). Of these five, one (lead) is classified as toxic to fertility and sexual function category 1A, two are classified as toxic to fertility and sexual function category 2 (acrylamide and cadmium). The other two are classified as toxic to fertility and sexual function category 1B (ethylene oxide and benzo[a]pyrene).

Furthermore, other substances included in tobacco are classified as reprotoxic category 1 (carbon monoxide) and category 2 (styrene and toluene) for their reprotoxic effects during embryonic and fetal development. However, the main target of this manuscript is fertility and we do not include these substances in our assessment.

### 3.2. Hazard Assessment and Systemic DNEL Estimation for the Substances Classified as Toxic to Fertility and Sexual Function in Reference Cigarette 1R6F

#### 3.2.1. Acrylamide

In 2005, the National Toxicology Program Center for the Evaluation of Risks to Human Reproduction (NTP-CERHR) published a monograph summarizing and evaluating the available evidence on the reproductive toxicity of acrylamide [10]. The main studies on fertility are summarised in Table 4. Of the six different studies summarized by the NTP-CERHR, the NOAEL ranged from 2 to 30 mg/kg bw/day. The LOAEL ranged from 5 to 45 mg/kg bw/day for the studies of different durations, with the most frequently reported critical effect being a reduced live litter size.

Acrylamide will be inhaled in the gaseous fraction and will be easily absorbed in the bloodstream via the lung tissue. Furthermore, some studies indicate that the detoxification pathways of acrylamide do not have impact on the substance deposition at doses relevant to human toxicity [11], therefore the internal dose from the whole body can be extrapolated to reproductive tissues.

The REACH registration dossier for acrylamide states that, in the case of oral exposure in mice, the excreted dose ranges from 53% to 79% [12]. Thus, in the most extreme case, the oral absorption of acrylamide in mice is 79%. However, for the purpose of calculating the most conservative DNEL, we used an oral absorption value of 53%.

The systemic DNELs for fertility and sexual function derived from the information summarised in Table 4 were estimated using the uncertainty factors presented in Table 1 and Table 2. The calculations are shown in Table 5.

Therefore, using the most conservative approach, a systemic DNEL (the internal dose of acrylamide that should not result in impaired fertility or sexual function) of 4.7 µg/kg bw/day was taken.

#### 3.2.2. Benzo[a]pyrene

The USEPA reviewed the toxicity of benzo[a]pyrene in 2017 [13]. The main hazards reviewed are developmental and reproductive toxicity, immunotoxicity and carcinogenicity. The main studies on fertility and sexual function are summarised in Table 6. Effects on male fertility and sexual function are observed after repeated oral and inhalation exposure; the most notable effects are changes in sperm parameters, reduced weight of reproductive organs, hormonal changes, testicular lesions and impaired fertility [13]. Female fertility and sexual function are also altered after exposure to benzo[a]pyrene by both oral and inhalation routes. Studies in rats and mice report fertility-related effects, including reductions in ovarian follicle populations, serum oestradiol and fertility [13].

The toxicological profile of polycyclic aromatic hydrocarbons (PAHs) reviewed by the Agency for Toxic Substances and Disease Registry (ATSDR) in 1995 states that the pulmonary absorption rate of benzo[a]pyrene is influenced by the solubility of the vehicle [14]. Specifically, approximately 70% of benzo[a]pyrene is reported to be excreted 6 h after intratracheal instillation with triethylene glycol [14]. The excretion rates are 58.4% and 56.2% with ethyl laurate and tricaprylin as vehicles, respectively, for the same 6-h period [14]. To be conservative and to accommodate for the effect of the vehicle, we estimated the systemic DNEL using inhalation studies with a default absorption rate of 56%. Oral absorption of benzo[a]pyrene in rats is not complete and appears to be influenced by oils and fats in the gastrointestinal tract [14]. Oral absorption of benzo[a]pyrene in rats after high dietary or gavage exposure is estimated to be 38–58% [14]. Again, based on the worst reasonable case, we used the lowest reported absorption rate (38%) to set the systemic DNEL using oral studies.

Thus, we estimated systemic DNELs for fertility and sexual function using the oral and inhalation studies summarised in Table 6 and the default physiological parameters and UF summarised in Table 1 and Table 2. The estimated DNELs derived from the oral toxicity studies are shown in Table 7. The estimated DNELs derived from the inhalation toxicity studies are shown in Table 8.

The DNELs estimated from oral toxicity studies ranged from 0.72 to 109 µg/kg bw/day (720 and 1.1 × 10^5^ ng/kg bw/day) (Table 7). They were significantly higher than the DNELs derived from inhalation toxicity studies, which ranged from 36 to 108 ng/kg bw/day (Table 8). Therefore, based on the worst reasonable case, we used the lowest DNEL of 36 ng/kg bw/day for our assessment (shaded in Table 8). The use of inhalation studies also covers for the particulate fraction of the substance that will become available into the systemic circulation, considering that deposition fraction in the lower respiratory tract will be higher than the of the vapour fraction [15].

#### 3.2.3. Cadmium

The REACH registration dossier for cadmium includes six different key studies and two additional supporting studies on fertility and sexual function toxicity [16]. Two of the six key studies were inhalation studies and the remaining four were oral exposure studies. One of the inhalation studies was conducted in mice and showed no changes in fertility or sexual function at the highest dose level. A second study in mice by oral route obtained the same conclusion. It was therefore unreliable for setting a DNEL and was not included in our assessment. Therefore, this study was not usable for our purposes. Table 9 summarises the most important studies. The reported effects were reduced sperm count, longer oestrous cycles and fewer copulations, pregnancies, implantations and live foetuses.

The same REACH registration dossier for cadmium states that gastrointestinal absorption is usually less than 5%, while inhalation absorption ranges from 10–30% for dust to 25–50% for fumes [16]. These figures are similar to those reported by the WHO [17]. Based on this information, we took a conservative approach and used absorption rates of 5 and 10% for the oral and inhalation routes, respectively, to set DNELs for fertility and sexual function according to cadmium exposure. The estimated DNELs based on oral toxicity studies are presented in Table 10. The estimated DNELs based on inhalation toxicity studies are shown in Table 11.

The estimated DNELs from oral toxicity studies ranged from 0.17 to 4.8 µg/kg bw/day (170 to 4800 ng/kg bw/day) (Table 10). They were significantly higher than the DNELs obtained from inhalation toxicity studies, which ranged from 14 to 274 ng/kg bw/day (Table 9). Therefore, based on the worst reasonable case, we used the lowest DNEL of 14 ng/kg bw/day (shaded in Table 11) for our assessment. The use of an inhalation study for this substance covers for the particulate fraction of cadmium that will become available into the systemic circulation, also considering the distribution and deposition of the substance in the lung as main organ [18].

#### 3.2.4. Ethylene Oxide

The REACH registration dossier for ethylene oxide contains three different studies with relevant toxicological information on fertility [19]. In particular, this dossier includes two sub chronic inhalation toxicity studies reporting testicular degeneration and lower absolute testis weights. One study on reproductive effects in rats exposed to ethylene oxide by inhalation for one generation reports a reduction of the number of pups per litter. Table 12 summarises these studies.

Ethylene oxide is highly soluble in the blood and therefore pulmonary uptake is expected to be rapid and dependent on the ventilation rate and the concentration of ethylene oxide in the atmosphere. The inhalation absorption rate is 100% according to the REACH registration dossier for ethylene oxide [24], and is also supported by the WHO [20]. Therefore, we assumed 100% lung uptake for the estimation of ethylene oxide DNELs from the studies summarised in Table 12. This assumption covers for the uncertainty regarding the gaseous fraction that will be inhaled. The systemic DNEL was set at the lowest value reported in Table 13, which is 0.37 mg/kg bw/day (370 µg/kg bw/day).

Regarding the availability of the substance in the reproductive tissue, Filser et al. [21] reported a blood: air partition coefficient of 61 of ethylene oxide for humans, while for reproductive tissues the partition coefficient available in rat testes, has a value of 83 [22], indicating a rapid perfusion into this tissue.

##### 3.2.5. Lead

In their analysis of the constituents of reference cigarettes, Jaccard [5] reported the presence of lead below the limit of quantification in some replicates, while in other determinations an amount of 28.1 ± 0.6 ng lead/cigarette was reported. Since lead and its compounds are classified as reprotoxic to humans (Category 1A) and labelled as “*may damage fertility and the unborn child*” in the ECHA Classification and Labelling Inventory [8] we decided to include lead in our assessment and derived a DNEL as described below.

The REACH registration dossier for lead contains three different studies on fertility toxicity [23]. In the key study, rats (males only) were given 0.25, 0.5 and 1 g lead/L in the drinking water for 60 days (Table 14). A dose of 0.5 g/L resulted in a decrease in spermatid count and a reduction in the diameter of the seminiferous tubules, while at 1 g/L more severe adverse effects were observed, such as spermatogenic inhibition and Leydig cell atrophy. In a second study, mice were exposed to 0.5% lead in the drinking water from day 1 of intrauterine life until day 60 after birth and mating of these exposed animals resulted in a reduced number of implants and offspring with reduced litter size (Table 14). Finally, a third study reported that male rats exposed to 0.3% lead in their drinking water for up to 60 days had reduced sperm concentrations and sperm with reduced fertility (Table 14).

The REACH registration dossier for lead [23] includes a toxicokinetic study in adult monkeys. In this study, it was estimated that 26% of the oral absorption occurred when monkeys were dosed with lead by gavage. Another toxicokinetic experiment in rats showed that the intestine absorbed about 23% of the lead administered by gavage [3]. Again, as a worst-case scenario, we will use the lowest reported absorption rate (23%) to derive a systemic DNEL from oral studies.

Regarding the internal dose of lead in the body, approximately 95% of lead in adult tissues resides in mineralized tissues such as bone and teeth [25]. Concentrations of lead in testes or in prostate gland are 4 to 5 times lower than in kidney or liver [26], showing less accumulation and indicating that internal dose from studies on whole body can cover for the internal exposure in reproductive tissues.

The estimated DNELs based on animal data ranged between 11.5 and 69 µg/kg bw/day (Table 15). Therefore, a systemic DNEL (the internal dose of lead that should not result in impaired fertility) from animal data of 11.5 µg/kg bw/day should be considered as the most conservative option based on animal data.

Additionally, ATSDR developed a toxicological profile for lead summarising the major epidemiological findings regarding lead toxicity to fertility in humans [27]. These epidemiological studies are not designed to determine doses without effects, but they usually divide populations in and set percentiles. It is therefore challenging to derive no effect concentrations from the existing human data, and we decided to stick to animal data when characterising the risk to lead exposure.

The assessment above includes an extra uncertainty factor of 3, considered for covering uncertainties of extrapolation from oral to inhalation route. This safety factor can cover the uncertainties related to the inhalation of lead in the particulate fraction, as expected for this substance.

### 3.3. Exposure Assessment

The exposure assessment was carried out as explained in Section 2.4.3 and is presented in Table 16. The REACH registration dossier for acrylamide states that after inhalation exposure in rats, 31% of the dose is excreted in the urine, while 56% remains in the body and bioaccumulates in different tissues (blood, testes, skin, liver, kidneys, brain, spleen, lung and epididymis) [12]. To estimate a conservative exposure, we used an absorption rate of 87%, resulting from the addition of excreted acrylamide to the accumulated acrylamide. For lead, approximately 25% of inhaled lead chloride and lead hydroxide with mass median aerodynamic diameters of 0.26 and 0.24 µm, respectively, were deposited in the respiratory tract in adult humans, according to ATSDR [27]. Thus, to estimate the systemic exposure to lead from cigarette smoking, an absorption rate of 25% is assumed. For the other chemicals, the lung absorption used in the exposure assessment was the one used in the hazard assessment (Section 3.2).

### 3.4. Risk Characterisation

The risk characterisation was carried out as indicated in Section 2.4.4. Table 17 shows the estimated risk ratios for each substance.

## 4. Discussion

This work assesses the risk of alterations in fertility resulting from exposure to five different substances (acrylamide, benzo[a]pyrene, cadmium, ethylene oxide and lead) found in tobacco smoke and classified as reproductive toxicants of category 1A, 1B or 2 (Table 3).

### 4.1. Mechanism of Toxicity

#### 4.1.1. Acrylamide

The mechanism of reproductive toxicity of acrylamide involves interference of the substance with the kinesin-related motor proteins, which are important in sperm motility and could alter reproduction parameters [28]. Furthermore, acrylamide has shown to cause severe degeneration of ovaries and testicular epithelial tissues in rats [29].

#### 4.1.2. Benzo[a]pyrene

Exposure to PAHs has been associated with higher levels of PAH-DNA adducts in sperm and male infertility [30]. Furthermore, an increased sperm DNA fragmentation, analysed by sperm chromatin structure assay, and an increase of immature sperm cells in humans were significantly associated with exposure to benzo[a]pyrene, as observed by Rubes et al. [31]. These authors concluded that short-term exposure to 1 ng benzo[a]pyrene/m^3^ significantly increases DNA fragmentation in the mature sperm. Both oral and inhalation exposure of rodents to benzo[a]pyrene has been shown to lead to decreased epididymal sperm motility and altered morphology [32,33,34].

Regarding female fertility, several mechanistic studies, both in vivo and in vitro [35,36,37] suggest that benzo[a]pyrene impacts fertility through the disruption of folliculogenesis. This finding is indirectly supported by observations of premature ovarian senescence in women exposed to cigarette smoke [38].

#### 4.1.3. Cadmium

Cadmium reproductive toxicity is mediated by multiple mechanisms, including structural damage to testis vasculature and blood–testis barrier, inflammation within the testis, cytotoxicity on Sertoli and Leydig cells, oxidative stress mainly by means of mimicry and interference with zinc and selenium and apoptosis within the testis. Furthermore, experimental studies in animals and evidence from human subjects exposed to cadmium suggest that its mechanisms of reprotoxicity might be linked to downstream signalling pathways, such as the ubiquitin proteasome system signalling pathway, and the calcium and cyclic AMP pathways [39].

#### 4.1.4. Ethylene Oxide

ECHA’s Risk Assessment Committee indicated that, since ethylene oxide is a well-established mutagen, it is possible that effects observed in one-generation studies, such as post-implantation losses and decreased incidence of implantations are mediated by a genotoxic mechanism [40]. In particular, post-implantation losses observed during the pre-mating period could be due to dominant lethal effect caused by a genotoxic insult. Genotoxic insult during specific stages of spermatogenesis may also affect sperm quality by increasing the number of abnormal sperm as suggested by Ribeiro et al. [41]. Nevertheless, other mechanisms of reproductive toxicity could not be excluded.

#### 4.1.5. Lead

It is still unclear whether the reprotoxicity of lead is a direct effect on reproductive organs, on the endocrine control of reproduction, or whether it consists of a combination of both [42]. There is doubt in the scientific community about the importance of lead deposition in the tubules of the testis and whether the localisation or distribution of lead in spermatozoa determines its possible adverse effect. A hypothesis about the blood–testis barrier (functionally very similar to the blood- brain barrier) suggests that the germinal epithelium is divided into two compartments: the basal compartment, related to spermatogonia, and the adluminal compartment, mainly related to more differentiated cells. Substances, either essential or toxic could reach the first compartment more easily and seem to be excluded from the second by occlusive junctions. Therefore, this could imply an easier interaction between lead and germinal cells than the interaction with differentiated cells [43].

#### 4.1.6. Overall Mechanism of Toxicity

These substances can cause reproductive effects via multiple mechanisms. Acrylamide and cadmium have a mode of action that can cause structural damage to reproductive organs, impairing their function and reducing fertility. Benzo[a]pyrene and ethylene oxide are known to cause DNA fragmentation, leading to mutations in germinal cells. Other mechanisms, such as hormonal disruption that could interfere with ovulation or sperm production, caused by exposure to these substances cannot be disregarded. Nevertheless, there is currently lacking information whether these substances could activate similar pathways.

### 4.2. Risk Assessment for Threshold Mechanisms of Toxicity

#### 4.2.1. Exposure to Individual Substances

Risk was assessed using the ratio of the systemic exposure (i.e., the internal dose produced by tobacco consumption) to the systemic DNEL (i.e., the internal dose that is predicted to cause no adverse effects and is considered safe). If the risk ratio is greater than 1, the exposure exceeds the safe dose, and adverse effects are possible. Conversely, a risk ratio below 1 means that the safe dose is higher than the exposure and adverse effects are not expected.

In our risk characterisation, the risk ratio for all the fives substances evaluated were well below 1, ranging from 0.00002 for lead to 0.23 for acrylamide. Therefore, none of the five analysed substances would be expected to cause significant fertility alterations individually at the doses resulting from the consumption of 20 cigarettes per day, even in the smoking regime defined as intensive by the International Standard Organization (ISO) [6].

Jaccard and co-workers [5] quantified up to 47 different substances in the smoke of a reference cigarette. They also reported the presence of substances below the limit of quantification, such as arsenic, chromium, nickel, selenium, nitrobenzene and resorcinol. From this short list, it should be noted that nitrobenzene is also classified as category 1B reprotoxic substance [8] for fertility effects. However, it is not possible to make an assessment of all these substances, as quantitative information on their potential exposure is lacking, and therefore the possibility that these substances could contribute to affecting fertility and sexual function in smokers cannot be ruled out.

It should also be noted that not all of the substances listed in Table 3 have a harmonised classification, and for those with a non-harmonised classification may be other reprotoxic substances that have not yet been identified. Therefore, the risk cannot be conveniently assessed. Consequently, and in order to have a better overview of the reproductive toxicity effects of the components of the mixture, we have compiled in Supplementary Table S1, some reproductive toxicity data (NOAEL and LOAEL for reproductive toxicity and NOAEL for parental toxicity) for the substances present in the mixture that do not have a harmonised classification for reproductive toxicity. This information has been collected from REACH registration dossier database of ECHA, allowing to identify whether any reproductive effects have been observed at doses not causing parental toxicity.

This information shows that for most of the substances present in the tobacco mixture there is no available data on their reproductive toxicity effects. From those substances where there is some information available on this endpoint, the vast majority have values of NOAEL for parental toxicity that are lower than the NOAEL values for reproductive toxicity. This may indicate that the substance might show the reproductive toxicity effects once parental adverse effects have been shown. In a couple of substances, the NOAEL values for parental and reproductive toxicity are the same. Nevertheless, the information available does not allow for a quantitative assessment and it is presented only for information purposes but suggests that it is unlikely that some of these substances could be responsible of the fertility impairments detected in epidemiological studies with smoker populations.

Similarly, it has been known since at least 1968 [44] that the number of substances in tobacco smoke is at least 1200, of which the majority are not considered to be those reported in the reference cigarette 1R6F. It is therefore plausible that some of this long list of substances could be toxic to fertility.

We assessed exposure for smokers of 20 cigarettes a day. However, EUROSTAT points out that 5.9% of people reported as smokers consume at least 20 cigarettes a day or more [45]. For these people, the exposure would be higher than we estimated, and the risk could therefore be closer to 1. The risk for these heavy smokers could be easily estimated by applying the appropriate corrections for cigarette consumption for exposure estimation according to the formula shown in Section 2.4.3.

#### 4.2.2. Exposure to Mixtures

This work assesses the risk of impairment of fertility via the threshold mechanisms of cadmium, ethylene oxide, acrylamide, lead and benzo[a]pyrene individually. In Table 17 we also indicate the risk of the mixture, considering a cumulative effect of the substances when their individual risk ratios are summed up. Taking this approach, the cumulative risk of the mixture is estimated to be 0.48. However, the information provided in this manuscript cannot rule out the possibility that toxicity to fertility and sexual function may be exacerbated by synergism due to exposure to multiple components in a complex mixture such as tobacco smoke. Indeed, it is possible that co-exposure to any of the components (not necessarily described as reproductive toxicants) could in some way increase the bioavailability of any of the five assessed substances that are present in the mixture. In each of these situations, the risks ratios estimated in Table 17 could be significantly higher and it would be then difficult to determine whether or not the DNEL would be exceeded by the exposure.

Benzo[a]pyrene (Table 6) and ethylene oxide (Table 12) are reported to be toxic for male fertility and cadmium for female fertility (Table 9). Although this seems unlikely due to the low- risk ratio in Table 17, especially for ethylene oxide, an enhancer effect of these substances cannot be excluded if both members of the couple (man and woman) are tobacco users and have their respective fertility impaired.

Nevertheless, considering the risk ratios estimated in this evaluation, both individually and for the whole smoke mixture, there is conflicting conclusions from the risk assessment point of view when trying to explain the evidence stemming from the epidemiological data.

#### 4.2.3. Synergistic Effects

The main metabolic pathway of acrylamide is conjugation with glutathione, which accelerates urinary excretion. There is also an alternative pathway that converts acrylamide to glycidamide, a substance whose toxicity to fertility and sexual function has not been well studied, although there is some information suggesting that it may be at least as toxic as acrylamide [10,46,47]. The 2E1 isoform of cytochrome P450 is involved in the biotransformation of acrylamide to glycidamide in mice [10], and it is unknown which isoform is involved in humans.

It is well known that the expression of cytochrome P450 is induced by exposure to various xenobiotics. In the case of human enzymes, the induction of isoforms 1A1, 1B1, 1A2 and 2S1 has been demonstrated by polycyclic aromatic hydrocarbons; the induction of isoform 2A6 by heavy metals such as cadmium and the induction of isoform 2E1 (the same responsible for the biotransformation of acrylamide in mice) by benzene derivatives such as styrene and toluene [48]. It is noteworthy that the presence of these inducers (cadmium, polycyclic aromatic hydrocarbons such as benzo[a]anthracene and benzo[a]pyrene, styrene, benzene and toluene) has been demonstrated in cigarette smoke (Table 3).

In conclusion, it seems plausible that the level of expression of 2E1 (and other isoforms) of cytochrome P450 was increased in smokers due to chronic exposure to some inducers found in cigarette smoke. These individuals may have increased the metabolism of acrylamide to glycidamide to the detriment of the conjugation of acrylamide with glutathione; this could prolong the presence of substances toxic to fertility in the body. Thus, the possibility of synergistic effects between acrylamide and some other substances present in tobacco cigarettes cannot be ruled out.

### 4.3. Route-to-Route Extrapolation

The critical studies for deriving DNELs for cadmium, benzo[a]pyrene and ethylene oxide were inhalation studies and therefore no route-to-route extrapolation is needed. On the contrary, for acrylamide no inhalation toxicity study was found and therefore an oral-to-inhalation extrapolation had to be performed, which inevitably leads to uncertainty.

One of the reasons for this uncertainty is the possibility of first-pass metabolism. It has been reported that acrylamide is rapidly detoxified and eliminated by conjugation with glutathione, the second major metabolite being glycidamide [10]. Glycidamide is not classified as a reproductive toxicant, but it was found that intraperitoneal injection of 50 mg glycidamide/kg bw/day for 14 days in rats resulted in reduced vas deferens sperm count, testicular protein content, epididymal weight and vas deferens sperm viability [49]. The same treatment with acrylamide caused reduced vas deferens sperm count but not all the other effects described for glycidamide [49]. Thus, the limited evidence suggests that glycidamide may be a reproductive toxicant that impairs male fertility to a greater extent than the parent acrylamide. This is also supported by other in vitro studies suggesting that glycidamide induces oxidative stress and apoptosis in mouse Leydig and Sertoli cells [46]. There is also further evidence of glycidamide’s ability to affect female fertility, as it can induce mouse oocyte degeneration in vitro [47].

Therefore, the extrapolation from oral to inhalation might underestimate the derived DNEL if glycidamide were considered to be toxic for fertility. However, we do not believe that this underestimation could significantly change the outcome of the assessment because glycidamide is not the major metabolite of acrylamide and because it is also rapidly biotransformed and detoxified by conjugation with glutathione and hydrolysis by epoxide hydrolase [10].

The Guidance on route-to-route extrapolation of toxicity data in the health risk assessment of chemicals notes that in the case of oral to inhalation extrapolation, this particular route-to-route extrapolation has a high risk of underestimating toxicity due to differences in the factors that govern pulmonary and oral absorption [50]. This guidance provides default values for oral to respirable extrapolation that can be considered conservative. These default values are 50% of oral absorption for substances of high toxicity (as in the case of acrylamide) and 100% of respirable absorption [50]. In our assessment we use very similar absorption values, namely 53% and 87% for oral and inhalation respectively. Therefore, our absorption values could be considered conservative and it is unlikely that they could contribute significantly to an underestimation of the toxicity of acrylamide in this extrapolation.

### 4.4. Risk Assessment for Non-Threshold Mechanisms of Toxicity

Three of the substances analysed in this work (acrylamide, benzo[a]pyrene and ethylene oxide) are considered to be substances known to induce heritable mutations or may be expected to induce heritable mutations in human germ cells and are classified as Muta 1B. Cadmium is considered a substance of concern to man because it may induce heritable mutations in human germ cells and is classified as Muta 2 (Table 3). However, it is also noteworthy that several cadmium salts, such as nitrate, hydroxide or carbonate, are also classified as Muta 1B [8].

The genotoxic activity of benzo[a]pyrene is well established both in vitro and in vivo [14]. In vitro, benzo[a]pyrene has been shown to induce gene mutations in somatic cells, DNA adduct formation, chromosome damage in germinal and somatic cells, unscheduled DNA synthesis and sister chromatid exchange in mammalian cells. In human cells, binding to DNA causing gene mutations, sister chromatid exchange, chromosome aberrations and unscheduled DNA synthesis have also been reported [14]. In vivo studies indicate that effects similar to those reported in vitro are also observed in intact animals and that benzo[a]pyrene is, therefore, genotoxic in both somatic and germinal cells.

In vitro genotoxicity studies with acrylamide show that the substance is unable to induce gene mutations in four different strains of *Salmonella typhimurium* and in Chinese hamster ovary cells, and induces chromosomal aberrations in Chinese hamster V79 lung fibroblasts. In rodents, a dominant lethal assay gave positive results, possibly due to chromosome deletions [12].

The REACH registration dossier for ethylene oxide [19] reports that this substance induces mutations in two different *Salmonella typhimurium stains*, in vitro sister chromatid exchange in human peripheral lymphocytes, in vitro gene mutations in Chinese hamster lung fibroblasts and in vitro cell transformation of C3H/10T1/2 mouse embryo fibroblasts. Ethylene oxide is positive in the male mouse dominant lethal assay. It causes translocations in mouse somatic and germ cells and chromosomal aberrations in mice. It is noteworthy that several of these positive in vivo results were obtained by inhalation exposure, which appears to be the most relevant route of exposure to ethylene oxide through tobacco consumption. Overall, there is overwhelming evidence that ethylene oxide is genotoxic to germ cells.

Several forms of cadmium are classified as either Muta 1B or Muta 2 depending on their solubility. Cadmium chloride induces genotoxicity, as evidenced by positive results in chromosome aberration, gene mutation and micronucleus assays in cultured mammalian cells [51,52,53]. Exposure to cadmium chloride by inhalation causes a positive response in the comet assay of mouse testicular DNA and in liver, kidney, bone marrow and brain cells [51,52,53]. An in vivo mouse spermatogonial chromosome aberration assay has also confirmed the mutagenicity of cadmium salts to germ cells [51,52,53]. Overall, there is sufficient evidence that systemically available cadmium is a mutagenic hazard to germ cells in animals.

It should also be noted that the total particulate matter of the reference cigarette 1R6F, produced in an intensive smoking regime, gave positive results in the Ames test with *Salmonella typhimurium* strains TA98 (both with and without exogenous metabolic activation), TA100 and TA137 (both with and without exogenous metabolic activation) and was also positive in the in vitro micronucleus test [5]. These results indicate a remarkable risk of genotoxicity to germ cells under in vivo physiological conditions.

Apart from these four substances classified as toxic to fertility and sexual function, of the 47 different substances quantified in tobacco smoke, three others (1,3-butadiene, benzene and propylene oxide) are considered mutagenic (classified as category 1B) and eight (acetaldehyde, pyrocatechol, crotonaldehyde, formaldehyde, hydroquinone, isoprene, phenol and quinoline) are suspected mutagens (classified as category 2) (Table 3). This mutagenicity classification refers to the ability of the classified substances to alter the genetic material of germ cells. Therefore, there is a possibility that some of these 15 substances, even those not classified as toxic for reproduction, may cause genetic damage in oocytes and/or spermatozoa, thereby affecting fertility. Furthermore, as mutagenesis is a threshold free change in toxicity, there would be no safe dose. This means that even if a dose–response relationship is observed, some proportion of the exposed population could always experience adverse effects.

### 4.5. Comparison with Findings from Other Published Studies

#### 4.5.1. Animal Studies

We reviewed the literature for animal studies that evaluated the effect of tobacco smoke on fertility and found little reliable information that could be directly compared with our study for several reasons, such as very old studies, inappropriate routes of exposure (i.e., intraperitoneal injection of tobacco smoke condensate), endpoints based on changes in molecular pathways of male and female reproductive organs rather than fertility. In all of these cases, the information is not suitable for comparison with our study, which was designed to address risk rather than adverse outcome pathways.

#### 4.5.2. Epidemiological Studies

The WHO considers infertility as a male and female disease, defined as the failure to achieve pregnancy after at least 12 months of regular unprotected sexual intercourse. The WHO has addressed the magnitude of the problem, concluding that the lifetime prevalence (the proportion of a population who have ever experienced infertility in their lifetime) is 17.5% (95% confidence interval (95%CI) = 15.0–20.3), and that this prevalence is similar across countries with different income levels [4].

Sharma [54] analyzed 20 studies with 6150 participants (2757 smokers and 3393 non-smokers) and concluded that exposure to cigarette smoking was associated with low sperm count (−9.72 × 10^6^/mL; 95%CI = −13.32 to −6.12 × 10^6^/mL) and reduced motility (−3.48%; 95%CI = −5.53 to −1.44%). Other authors have also concluded that tobacco smoking impairs semen quality, probably via oxidative stress [55,56]. The Practice Committee of the American Society for Reproductive Medicine (ASRM) [57] concluded that there is reasonable evidence that smoking affects sperm parameters, but whether it has a real effect on male fertility remains to be established.

The success of fertility treatments in women who smoke appears to be significantly lower than in non-smoking women. A meta-analysis that analyzed information from 26 different studies found a significant decrease in the live birth rate per cycle for smoking patients (odds ratio (OR) = 0.59, 95%CI = 0.44–0.79), a significantly lower clinical pregnancy rate per cycle for smoking women (OR = 0.53, 95%CI = 0.41–0.68) and a significant increase in the spontaneous miscarriage rate (OR = 2.22, 95%CI = 1.10–4.48) for smoking women [57]. A Danish cohort of 1456 women concluded that the adjusted risk of having a live birth was 1.18 (95%CI = 0.69–2.02) for self-reported smokers compared with non-smokers [58]. The Practice Committee of the ASRM [57] also concluded that there is good evidence that smokers require almost twice as many in vitro fertilizations attempts to achieve pregnancy as non-smokers.

## 5. Conclusions

We have evaluated the possibility that tobacco affects fertility and sexual function from the perspective of a risk assessment of the substances contained in tobacco and classified as reproductive toxicants for their effects on fertility. The changes in fertility resulting from tobacco exposure cannot be explained by the individual or mixed threshold effects of acrylamide, benzo[a]pyrene, cadmium ethylene oxide or lead. The possibility that the fertility impairments reported in epidemiological studies [54,55,56,58,59] were due to non-threshold molecular mechanisms of toxicity should be further explored.

In addition, from the substances quantified in tobacco smoke in Table 3, 12 are classified as category 1A or 1B carcinogens (i.e., human carcinogens) and four as category 2 carcinogens (i.e., suspected carcinogens). Although these substances do not necessarily affect the cells involved in fertility, they expose the organism to a series of alterations that require mechanisms of compensation and homeostasis (apoptosis, autophagy, repair of damaged DNA, etc.), which could require energy expenditure and hinder the production of fully functional oocytes and spermatozoa or other cells involved in reproduction as uterus.

Overall, the data presented in this paper suggest that it is not possible to establish a robust, scientifically based threshold level of tobacco use for fertility and sexual function risk management, and therefore tobacco should not be consumed in people seeking pregnancy. This recommendation applies to both active and passive smokers, because although it could be argued that the exposure of passive smokers is lower than that of active smokers, since no threshold or safe dose has been established, the pregnancies of passive smokers could also be at risk.

## Figures and Tables

**Table 1:** Standard physiological parameters according to the principle of allometric scaling. Data taken from [9].

Physiological Parameter	Human	Rat	Mice
Weight	70 kg	0.25 kg	0.03 kg
Respiratory rate	5 m^3^ air/6 h 0.2 L air/min/kg	0.8 L air/min/kg -	2.5 L air/h 1.4 L air/min/kg

**Table 2:** Uncertainty factors used to estimate a systemic DNEL. Data taken from [9].

Uncertainty Factor	Value
**Interspecies differences:**	
Allometric rat *	4
Allometric mouse *	7
Other differences	2.5
**Intraspecies differences:**	
General population (the most sensitive individuals)	10
**Extrapolation by duration:**	
A sub-chronic DNEL from a subacute starting value **	3
**Extrapolation when a critical value is a LOAEL**	3

* Not required for inhalation studies. ** Applied to all those studies with a duration of four weeks or less.

**Table 3:** Human health hazard classification of hazards for human health of the substances contained in the smoke of reference cigarette 1R6F. The substance list of the reference cigarette 1R6F was obtained from [5]. The classification was obtained from the ECHA Classification and Labelling Inventory. Substances classified as toxic for fertility and sexual function are shaded. LOQ = Limit of quantification. Muta. = Germ cell mutagenic. Carc. = Carcinogenicity. Acute Tox. = Acute toxicity. Skin Irrit. = Skin irritant. Eye Irrit. = Eye irritant. Skin Corr. = Skin corrosive. Skin Sens. = Skin sensitizer. Eye Dam. = Ocular corrosive. Asp. Tox = Toxic by aspiration. STOT SE = Toxic to any organ after a single exposure. STOT RE = Toxic to any organs after repeated exposure.

Substance	CAS Number	µg/Cigarette	Hazard Class and Category
1,3-butadiene	106-99-0	114	Muta. 1B Carc. 1A
1-naphthylamine	134-32-7	0.0267	Acute Tox. 4
2-naphthylamine	91-59-8	0.0162	Acute Tox. 4 Carc. 1A
3-aminobiphenyl	2243-47-2	0.00449	Acute Tox. 4 Skin Irrit. 2 Eye Irrit. 2 STOT SE 3
4-aminobiphenyl	92-67-1	0.003313	Acute Tox. 4 Carc. 1A
Acetaldehyde	75-07-0	1859	Eye Irrit. 2 STOT SE 3 Muta. 2 Carc. 1B
Acetamide	60-35-5	14	Carc. 2
Acetone	67-64-1	635	Eye Irrit. 2 STOT SE 3
Acrolein	107-02-8	173	Acute Tox. 2 Acute Tox. 3 Skin Corr. 1B Acute Tox. 1
Acrylamide	79-06-1	4.49	Acute Tox. 3 Acute Tox. 4 Skin Irrit. 2 Eye Irrit. 2 Skin Sens. 1 Acute Tox. 4 Muta. 1B Carc. 1B STOT RE 1 **Repr. 2**
Acrylonitrile	107-13-1	19.2	Acute Tox. 3 Acute Tox. 3 Acute Tox. 3 Skin Irrit. 2 Eye Dam. 1 Skin Sens. 1 Acute Tox. 3 STOT SE 3 Carc. 1B
Ammonia	7664-41-7	35.9	Skin Corr. 1B Acute Tox. 3
Benzene	71-43-2	82.3	Skin Irrit. 2 Eye Irrit. 2 Asp. Tox. 1 Muta. 1B Carc. 1A STOT RE 1
Benzo[a]anthracene	56-55-3	0.027	Carc. 1B
Dibenz[a,h]anthracene	53-70-3	0.000892	Carc. 1B
Benzo[a]pyrene	50-32-8	0.0138	Skin Sens. 1 Muta. 1B Carc. 1B **Repr. 1B**
Resorcinol	108-46-3	0.0018	Acute Tox. 4* Skin Irrit. 2 Eye Irrit. 2
Chromium	7440-47-3	Below LOQ	Skin Sens. 1 Resp. Sens. 1 Eye Irrit. 2 STOT SE 2 Carc. 2
Nickel	7440-02-0	Below LOQ	Skin Sens. 1 Carc. 2 STOT RE 1
Selenium	7782-49-2	Below LOQ	Acute Tox. 3 Acute Tox. 3 STOT RE 2
Arsenic	7440-38-2	0.00757	Acute Tox. 3 Acute Tox. 3
Lead	7439-92-1	0.00281	Lact. **Repr. 1A**
Cadmium	7440-43-9	0.0888	Carc. 1B STOT RE 1 Muta. 2 **Repr. 2** Acute Tox. 2
Pyrocatechol	120-80-9	91.9	Acute Tox. 3 Acute Tox. 3 Skin Irrit. 2 Eye Irrit. 2 Muta. 2 Carc. 1B
Carbon monoxide	630-08-0	29400	Acute Tox. 3 STOT RE 1 Repr. 1A
Crotonaldehyde	4170-30-3	55	Acute Tox. 3 Acute Tox. 3 Skin Irrit. 2 Eye Dam. 1 Acute Tox. 2 STOT SE 3 Muta. 2 STOT RE 2
Ethylene oxide	75-21-8	17.3	Acute Tox. 3 Skin Corr. 1 Eye Dam. 1 Acute Tox. 3 STOT SE 3 STOT SE 3 Muta. 1B Carc. 1B STOT RE 1 **Repr. 1B**
Formaldehyde	50-00-0	104	Acute Tox. 3 Acute Tox. 3 Skin Corr. 1B Skin Sens. 1 Acute Tox. 3 Muta. 2 Carc. 1B
Hydrogen cyanide	74-90-8	352	Acute Tox. 2 Acute Tox. 1 Acute Tox. 2
Hydroquinone	123-31-9	88.7	Acute Tox. 4 Eye Dam. 1 Skin Sens. 1 Muta. 2 Carc. 2
Isoprene	78-79-5	859	Muta. 2 Carc. 1B
m-Cresol	108-39-4	2.98	Acute Tox. 3 Acute Tox. 3 Skin Corr. 1B
Mercury	7439-97-6	0.00468	Acute Tox. 2 STOT RE 1 Repr. 1B
Methyl ethyl ketone	78-93-3	164	Eye Irrit. 2 STOT SE 3
N-nitrosoanabasine	37620-20-5	0.0244	Acute Tox. 3
N-nitrosoanatabine	887407-16-1	0.259	Acute Tox. 3
Nicotine	54-11-5	2000	Acute Tox. 2 Acute Tox. 2 Acute Tox. 2
1-Butanone, 4-(methylnitrosoamino)-1-(3-pyridinyl)-	64091-91-4	0.208	Acute Tox. 3 Skin Sens. 1 Carc. 2
N-nitrosonornicotine	80508-23-2	0.23	Acute Tox. 3 Carc. 2
Nitrogen monoxide	10102-43-9	329	Skin Corr. 1B Eye Dam. 1 Acute Tox. 1
NOx	-	405	Skin Corr. 1B Acute Tox. 2
o-Cresol	95-48-7	3.12	Acute Tox. 3 Acute Tox. 3 Skin Corr. 1B
o-Toluidine	95-53-4	0.109	Acute Tox. 3 Eye Irrit. 2 Acute Tox. 3 Carc. 1B
p-Cresol	106-44-5	7.77	Acute Tox. 3 Acute Tox. 3 Skin Corr. 1B
Phenol	108-95-2	12.5	Acute Tox. 3 Acute Tox. 3 Skin Corr. 1B Acute Tox. 3 Muta. 2 STOT RE 2
Propionaldehyde	123-38-6	119	Skin Irrit. 2 Eye Irrit. 2 STOT SE 3
Propylene oxide	75-56-9	1710	Acute Tox. 4 Acute Tox. 3 Eye Irrit. 2 Acute Tox. 3 STOT SE 3 Muta. 1B Carc. 1B
Pyrene	129-00-0	88	
Pyridine	110-86-1	36.4	Acute Tox. 4 Acute Tox. 4 Acute Tox. 4
Quinoline	91-22-5	0.427	Acute Tox. 4 Acute Tox. 4 Skin Irrit. 2 Eye Irrit. 2 Muta. 2 Carc. 1B
Styrene	100-42-5	20.4	Skin Irrit. 2 Eye Irrit. 2 Acute Tox. 4 STOT RE 1 (hearing organs) Repr. 2
Toluene	108-88-3	132	Skin Irrit. 2 Asp. Tox. 1 STOT SE 3 STOT RE 2 Repr. 2
Vinyl Chloride	75-01-4	0.109	Carc. 1A

**Table 4:** Acrylamide: Key studies to assess toxicity to fertility and sexual function. Data taken from [10].

Animals	Exposure	Critical Effect	Effect Level	Study ^1,2^
Male ddY mice	Drinking water: 21.3, 42.6, 64.0, 85.2 ppm; 4 weeks	Reduced litter size	NOAEL = 42.6 ppm (~9 mg/kg bw/day) LOAEL = 64.0 ppm (~14 mg/kg bw/day)	[1]
Male Long-Evans rats	Drinking water: 0, 50, 100, 200 ppm; 10 weeks	Impaired ejaculation, lower vaginal and uterine sperm and pregnancy rates	NOAEL = 50 ppm (about 4 mg/kg bw/day) LOAEL = 100 ppm (about 8 mg/kg bw/day)	[2]
Male Long-Evans rats	Gavage: 0, 15, 45 mg/kg bw/day; 5 days	Decreased oocyte fertilization	LOAEL = 15 mg/kg bw/day	[3]
Male Long-Evans rats	Gavage: 0, 5, 15, 30, 45, 60 mg/kg bw/day; 5 days	Decreased mating; fewer produced litters	NOAEL = 30 mg/kg bw/day LOAEL = 45 mg/kg bw/day	[4]
Male and female Swiss mice	Drinking water: 0, 3, 10, 30 ppm; 74 days	Reduced live litter size in two generations	NOAEL = 10 ppm (2.5 mg/kg bw/day) LOAEL = 30 ppm (8 mg/kg bw/day)	[5]
Male and female Fischer 344 rats	Drinking water: 0, 0.5, 2.0, 5.0 mg/kg bw/day; 2-generation reproduction toxicity assay	Reduced live litter size	NOAEL = 2 mg/kg bw/day LOAEL = 5 mg/kg bw/day	[6]

^1^ Note that we extracted the data in this table from the monograph and did not have access to the original reference. We present this information with the goal of allowing the reader to match each entry in this table with the specific study in the monograph that was used as a source of information. ^2^ [1] = Sakamoto and Hashimoto (full reference is missing in the monograph); [2] = Zenick et al., 1986; J Toxicol Environ Health 17: 457–472 (https://doi.org/10.1080/15287398609530840); [3] = Sublet et al., 1989; Toxicology 55: 53–67 (https://doi.org/10.1016/0300-483X(89)90174-1); [4] = Tyl (Full reference is missing in the monograph); [5] = Chapin et al., (full reference is missing in the monograph); [6] = Tyl et al., 2000; Reprod Toxicol 14: 385–401 (https://doi.org/10.1016/S0890-6238(00)00097-6).

**Table 5:** Estimation of the systemic DNEL for acrylamide. Critical values were taken from the referenced studies summarized in Table 4. Oral absorption was taken from the REACH registration dossier for acrylamide. Uncertainty factors were set according to the ECHA procedures for deriving DNELs for threshold endpoints [9] (Table 2). N = NOAEL. L = LOAEL. The lowest DNEL is shaded.

	[Study 1] ^1^		[Study 2] ^1^		[Study 3] ^1^		[Study 4] ^1^		[Study 5] ^1^		[Study 6] ^1^	
	N	L	N	L	L		N	L	N	L	N	L
Critical value (mg/kg bw/day)	9	14	4	8	15		30	45	2.5	8	2	5
Oral absorption	0.53	0.53	0.53	0.53	0.53		0.53	0.53	0.53	0.53	0.53	0.53
Transformation mg into µg	10^3^	10^3^	10^3^	10^3^	10^3^		10^3^	10^3^	10^3^	10^3^	10^3^	10^3^
*Uncertainty factors:*												
Interspecies allometric	7	7	4	4	4		4	4	7	7	4	4
Interspecies remaining	2.5	2.5	2.5	2.5	2.5		2.5	2.5	2.5	2.5	2.5	2.5
Intraspecies	10	10	10	10	10		10	10	10	10	10	10
LOAEL to NOAEL	1	3	1	3	3		1	3	1	3	1	3
Duration adjustment	3	3	1	1	3		3	3	1	1	1	1
Systemic DNEL (µg/kg bw/day)	9.1	4.7	21	14	8.8		53	27	7.6	8.1	11	8.8

^1^ See Table 4 for identification of studies.

**Table 6:** Benzo[a]pyrene: key studies to assess toxicity to fertility and sexual function. Data taken from [13].

Animals	Exposure	Critical Effect	Effect Level	Study ^1,2^
Male C57BL/6 mice	0, 1, 10 mg/kg bw/day, 42 days	Testicular changes Sperm alterations	LOAEL = 1 mg/kg bw/day	[7]
Male Hsd:ICR (CD1) mice	0, 1, 10, 50, 100 mg/kg bw/day; 60 days	Testicular changes Sperm alterations	NOAEL = 50 mg/kg bw/day LOAEL = 100 mg/kg bw/day	[8]
Male Sprague Dawley rats	0, 5 mg/kg bw/day; 84 days	Sperm alterations	LOAEL = 5 mg/kg bw/day	[9]
Male F344 rats	0, 75 µg/m^3^; 60 days; 4 h/day	Sperm motility	LOAEL = 75 µg/m^3^	[10]
Male F344 rats	0, 75 µg/m^3^; 60 days; 4 h/day	Testicular changes; testosterone and luteinizing hormone alterations	LOAEL = 75 µg/m^3^	[11]
Male Sprague Dawley rats	0, 5 mg/kg bw/day; 28 days	Testosterone level alterations	LOAEL = 5 mg/kg bw/day	[12]
Male Sprague Dawley rats	0, 1, 5 mg/kg bw/day; 90 days	Testosterone level alterations	NOAEL = 1 mg/kg bw/day LOAEL = 5 mg/kg bw/day	[13]
Female CD-1 mice	0, 10, 40 and 160 mg/kg bw/day; 10 days	Fewer females with viable litters	NOAEL = 40 mg/kg bw/day LOAEL = 160 mg/kg bw/day	[14]
Female Fisher 344	0, 50, 75, 100 µg/m^3^; 14 days; 4h/day	Reductions in pups born/litter and lower ovulation rate	NOAEL = 75 µg/m^3^ LOAEL = 160 µg/m^3^	[15]
Female Sprague Dawley rats	0, 2.5, 5 mg/kg bw/day; 60 days	Reduction ovary weight; ovarian granulosa cells apoptosis	NOAEL = 2.5 mg/kg bw/day LOAEL = 5 mg/kg bw/day	[16]
Female F344 rats	0, 25, 75, 100 µg/m^3^; 4 h/day; 10 days	Reductions in estradiol, prolactin and progesterone	NOAEL = 25 µg/m^3^ LOAEL = 75 µg/m^3^	[17]

^1^ Note that we extracted the data in this table from the monograph and did not have access to the original reference. We present this information with the goal of allowing the reader to match each entry in this table with the specific study in the monograph that was used as a source of information. ^2^ [7] = Mohamed et al., 2010; Hum Reprod 25: 2427–2433 (http://dx.doi.org/10.1093/humrep/deq205); [8] = Jeng et al., 2013; Environ Toxicol 30: 1–8 (http://dx.doi.org/10.1002/tox.21889); [9] = Chen et al., 2011; J Hazard Mater 186: 835–841 (http://dx.doi.org/10.1016/j.jhazmat.2010.11.078); [10] = Ramesh et al., 2008; Exp Toxicol Pathol 60: 269–280 (http://dx.doi.org/10.1016/j.etp.2008.02.010); [11] = Archibong et al., 2008; Int J Environ Res Public Health 5: 32–40 (http://dx.doi.org/10.3390/ijerph5010032); [12] = Chen et al., 2011; J Hazard Mater 186: 835–841 (http://dx.doi.org/10.1016/j.jhazmat.2010.11.078); [13] = Zheng et al., 2010; Toxicol Appl Pharmacol 248: 28–37 (http://dx.doi.org/10.1016/j.taap.2010.07.008); [14] = Mackenzie and Angevine 1981; Biol Reprod 24: 183–192 (https://doi.org/10.1095/biolreprod24.1.183); [15] = Archibong et al., 2012; Reprod Toxicol 34: 635–643 (http://dx.doi.org/10.1016/j.reprotox.2012.09.003); [16] = Xu et al., 2010; Toxicol Lett 199: 323–332 (http://dx.doi.org/10.1016/j.toxlet.2010.09.015); [17] = Archibong et al., 2002; Reprod Toxicol 16: 801–808 (https://doi.org/10.1016/S0890-6238(02)00058-8).

**Table 7:** Estimation of the systemic DNEL for benzo[a]pyrene from oral toxicity studies. Critical values were taken from the referenced studies summarized in Table 6. Oral absorption was taken from [14]. Uncertainty factors were set according to ECHA procedures for deriving DNELs for threshold endpoints [9] (Table 2). N = NOAEL. L = LOAEL.

	[Study 7] ^1^	[Study 8] ^1^		[Study 9] ^1^	[Study 12] ^1^	[Study 13] ^1^		[Study 14] ^1^		[Study 16] ^1^	
	L	N	L	L	L	N	L	N	L	N	L
Critical value (mg/kg bw/day)	1	50	100	5	5	1	5	40	160	2.5	5
Oral absorption rate	0.38	0.38	0.38	0.38	0.38	0.38	0.38	0.38	0.38	0.38	0.38
Transformation mg into µg	10^3^	10^3^	10^3^	10^3^	10^3^	10^3^	10^3^	10^3^	10^3^	10^3^	10^3^
*Uncertainty factors:*											
Interspecies allometric	7	7	7	4	4	4	4	7	7	4	4
Interspecies remaining	2.5	2.5	2.5	2.5	2.5	2.5	2.5	2.5	2.5	2.5	2.5
Intraspecies	10	10	10	10	10	10	10	10	10	10	10
LOAEL to NOAEL	3	1	3	3	3	1	3	1	3	1	3
Duration adjustment	1	1	1	1	3	1	1	3	3	1	1
Systemic DNEL (µg/kg bw/day)	0.72	109	72	6.3	2.1	3.8	6.3	29	39	9.5	6.3

^1^ See Table 6 for identification of studies.

**Table 8:** Estimation of the systemic DNEL for benzo[a]pyrene based on inhalation toxicity studies. Critical values were taken from the referenced studies summarized in Table 6. Inhalation absorption was taken from [14]. Uncertainty factors and physiological factors were set according to the ECHA procedures for deriving DNELs for threshold endpoints [9] (Table 1 and Table 2). The allometric factor is not applicable when setting an inhalation DNEL based on an inhalation animal study [9]. N = NOAEL. L = LOAEL. The lowest DNEL is shaded.

	[Study 10] ^1^	[Study 11] ^1^	[Study 15] ^1^		[Study 17] ^1^	
	L	L	N	L	N	L
Critical value (µg/m^3^)	75	75	75	160	25	75
Inhalation absorption rate	0.56	0.56	0.56	0.56	0.56	0.56
Transformation m^3^ into L	10^3^	10^3^	10^3^	10^3^	10^3^	10^3^
Transformation µg into ng	10^3^	10^3^	10^3^	10^3^	10^3^	10^3^
Exposure time (min/d)	240	240	240	240	240	240
Respiration rate (L/min/kg) (Table 1)	0.8	0.8	0.8	0.8	0.8	0.8
*Uncertainty factors:*						
Interspecies allometric	1	1	1	1	1	1
Interspecies remaining	2.5	2.5	2.5	2.5	2.5	2.5
Intraspecies	10	10	10	10	10	10
LOAEL to NOAEL	3	3	1	3	1	3
Duration adjustment	1	1	3	3	3	3
Systemic DNEL (ng/kg bw/day)	108	108	108	76	36	36

^1^ See Table 6 for identification of studies.

**Table 9:** Cadmium: key studies to assess toxicity to fertility and sexual function. Data taken from the REACH registration dossier for cadmium [16] available on the ECHA website.

Animals	Exposure	Critical Effect	Effect Level	Study ^1,2^
Male and female Fischer 344 rats	0, 25, 50, 100, 250 and 1000 µg/m^3^; 6 h/day, 5 days/week; 13 weeks	Increase in number of spermatids/testis Reduction in length of estrous cycle	NOAEL = 250 µg/m^3^ LOAEL = 1000 µg/m^3^	[18]
Female Wistar rats	0, 20, 160 and 1000 µg/m^3^; 5 h/day, 5 days/week; 20 weeks	↑ percentage of females with estrus cycles lasting over 6 d	NOAEL = 20 µg/m^3^ LOAEL = 160 µg/m^3^	[19]
Female Wistar rats	0, 0.04, 0.4, 4, 40 mg/kg bw/day; gavage; 14 weeks; 5 days/week	Length of estrous cycle was twice that of the control rats.	NOAEL = 4 mg/kg bw/day LOAEL = 40 mg/kg bw/day	[20]
Male and female Sprague Dawley	0, 0.1, 1, 10 mg/kg/day; 6 days/week; 3 weeks	↓ number of copulations, pregnancies, implants and live fetuses	NOAEL = 1 mg/kg bw/day LOAEL = 10 mg/kg bw/day	[21]

^1^ Note that we extracted the data in this table from the REACH registration dossier and did not have access to the original reference. We present this information with the goal of allowing the reader to match each entry in this table with the specific study in the monograph that was used as a source of information. ^2^ [18] = Key study report number 1 in [16] (reproductive toxicity section); [19] = Key study report number 3 in [16] (reproductive toxicity section) (Barański and Sitarek, 1987; Toxicol Lett 36(3):267–273 (https://doi.org/10.1016/0378-4274(87)90195-0)); [20] = Key study report number 5 in [16] (reproductive toxicity section) (Barański and Sitarek, 1987; Toxicol Lett 36(3):267–273 (https://doi.org/10.1016/0378-4274(87)90195-0)); [21] = Key study report number 6 in [16] (reproductive toxicity section) (Sutou et al., 1980; Ecotoxicol Environ Saf 4(1):51–56 https://doi.org/10.1016/0147-6513(80)90007-x)).

**Table 10:** Estimation of the systemic DNEL for cadmium from oral toxicity studies. Critical values were taken from the referenced studies summarized in Table 9. Oral absorption was taken from the REACH registration dossier for cadmium [16]. Uncertainty factors were set according to the ECHA procedures for deriving DNELs for threshold endpoints [9] (Table 2).

	[Study 20] ^1^		[Study 21] ^1^	
	NOAEL	LOAEL	NOAEL	LOAEL
Critical value (mg/kg bw/day)	4	40	1	10
Oral absorption rate	0.05	0.05	0.05	0.05
Transformation mg into µg	10^3^	10^3^	10^3^	10^3^
Adjustment to 7 day/week	5/7	5/7	7/7	7/7
*Uncertainty factors:*				
Interspecies allometric	4	4	4	4
Interspecies remaining	2.5	2.5	2.5	2.5
Intraspecies	10	10	10	10
LOAEL to NOAEL	1	3	1	3
Duration adjustment	1	1	3	3
Systemic DNEL (µg/kg bw/day)	1.4	4.8	0.17	0.56

^1^ See Table 9 for identification of studies.

**Table 11:** Estimation of the systemic DNEL for cadmium based on inhalation toxicity studies. Critical values were taken from the referenced studies summarized in Table 9. Inhalation absorption was taken from the REACH registration dossier for cadmium [16]. Uncertainty factors and physiological factors were set according to the ECHA procedures for deriving DNELs for threshold endpoints [9] (Table 1 and Table 2). The allometric factor is not applicable when setting an inhalation DNEL based on an inhalation animal study [9]. The lowest DNEL is shaded.

	[Study 18] ^1^		[Study 19] ^1^	
	NOAEL	LOAEL	NOAEL	LOAEL
Critical value (µg/m^3^)	250	1000	20	160
Inhalation absorption rate	0.1	0.1	0.1	0.1
Transformation m^3^ into L	10^3^	10^3^	10^3^	10^3^
Transformation µg into ng	10^3^	10^3^	10^3^	10^3^
Exposure time (min/day)	360	360	300	300
Respiration rate (L/min/kg) (Table 1)	0.8	0.8	0.8	0.8
Adjustment to 7 day/week	5/7	5/7	5/7	5/7
*Uncertainty factors:*				
Interspecies allometric	1	1	1	1
Interspecies remaining	2.5	2.5	2.5	2.5
Intraspecies	10	10	10	10
LOAEL to NOAEL	1	3	1	3
Duration adjustment	1	1	1	1
Systemic DNEL (ng/kg bw/day)	206	274	14	37

^1^ See Table 9 for identification of studies.

**Table 12:** Ethylene oxide: key studies to assess toxicity to fertility and sexual function. Data taken from the REACH registration dossier for ethylene oxide [19] available on the ECHA website. ↓ = Reduction.

Animals	Exposure	Critical Effect	Effect Level	Study ^1,2^
Male and female Fischer 344 rats	0, 90, 180, 270, 810 mg/m^3^; 6 h/day; 5 days/week; 8 weeks Subchronic inhalation toxicity study	testicular degeneration with abnormal spermatocytes and atrophy of seminiferous tubules, hypospermia, aspermia	NOAEL = 270 mg/m^3^ LOAEL = 810 mg/m^3^	[22]
Male and female B6C3F1 mice	18, 90, 180, 450 mg/m^3^; 6 h/day; 5 days/week; 10 weeks Subchronic inhalation toxicity study	Depressed absolute testicular weight	NOAEL = 180 mg/m^3^ LOAEL = 450 mg/m^3^	[23]
Male and female Fischer 344	18, 54, 180 mg/m^3^; 5 h/day; 5 days/week; 14 weeks One-generation reproductive toxicity	Reduction in number pups/litter	NOAEL = 54 mg/m^3^ LOAEL = 180 mg/m^3^	[24]

^1^ Note that we extracted the data in this table from the REACH registration dossier and did not have access to the original reference. We present this information with the goal of allowing the reader to match each entry in this table with the specific study in the monograph that was used as a source of information. ^2^ [22] = Supporting study report number 1 in [19] (repeated dose toxicity inhalation studies section); [23] = Weight of evidence study number 4 in [19] (repeated dose toxicity inhalation studies section) (Snellings et al., 1984 Toxicol Appl Pharmacol 76(3):510–518 (https://doi.org/10.1016/0041-008x(84)90355-7); [24] = Weight of evidence study in [19] (reproductive toxicity section) (Snellings et al., 1982 Toxicol Appl Pharmacol 63(3):382–388 https://doi.org/10.1016/0041-008x(82)90267-8).

**Table 13:** Estimation of the systemic DNEL for ethylene oxide. Critical values were taken from the referenced studies summarized in Table 12. Inhalation absorption was taken from the REACH registration dossier for ethylene oxide [19]. Uncertainty factors and physiological factors were set according to the ECHA procedures for deriving DNELs for threshold endpoints [9] (Table 1 and Table 2). The allometric factor is not applicable when setting an inhalation DNEL based on an inhalation animal study [9]. N = NOAEL. L = LOAEL. The lowest DNEL is shaded.

	[Study 22] ^1^		[Study 23] ^1^		[Study 24] ^1^	
	N	L	N	L	N	L
Critical value (mg/m^3^)	270	810	180	450	54	180
Inhalation absorption rate	1	1	1	1	1	1
Adjustment to 7 day/week	5/7	5/7	5/7	5/7	5/7	5/7
Transformation m^3^ into L	10^3^	10^3^	10^3^	10^3^	10^3^	10^3^
Exposure time (min/day)	480	480	480	480	300	300
Respiration rate (L/min/kg) (Table 2)	0.8	0.8	1.4	1.4	0.8	0.8
*Uncertainty factors:*						
Interspecies allometric	1	1	1	1	1	1
Interspecies remaining	2.5	2.5	2.5	2.5	2.5	2.5
Intraspecies	10	10	10	10	10	10
LOAEL to NOAEL	1	3	1	3	1	3
Duration adjustment	1	1	1	1	1	1
Systemic DNEL (mg/kg bw/day)	2.9	2.9	3.5	2.8	0.37	0.41

^1^ See Table 9 for identification of studies.

**Table 14:** Lead: key studies to assess toxicity to fertility and sexual function. Data taken from REACH registration dossier [23]. * Estimated using a default factor for sub-chronic studies in rats of 0.09 according to the guidance on selected default values to be used by the EFSA Scientific Committee [24]. ** Estimated using a default factor for sub-chronic studies in mice of 0.15 according to the guidance on selected default values to be used by the EFSA Scientific Committee [24]. These factors are used for converting substance concentrations in drinking water into daily doses in experimental animal studies.

Animals	Exposure	Critical Effect	Effect Level	Study ^1^
Male Albino rats	0, 0.25, 0.5, and 1 g/L in drinking water, 60 days	Decreases in seminiferous tubule diameter and spermatid count. Lumen of the seminiferous tubules filled with cellular debris	NOAEL = 0.25 g/L (22.5 mg/kg bw/day) * LOAEL = 0.5 g/L (45 mg/kg bw/day) *	[25]
Male and female NMRI mice	5 g/L (0.5%) in drinking water from day 1 of intrauterine life until 60 days after birth	Litter size and number of implants were significantly reduced.	LOAEL = 5 g/L (450 mg/kg bw/day) **	[26]
Male Sprague Dawley rats	3 g/L (0.3%) in drinking water for 14, 30, or 60 days	Decreased epididymal sperm concentrations. Sperm harvested from animals penetrated fewer eggs	LOAEL = 3 g/L (270 mg/kg bw/day) *	[27]

^1^ [25] = Key study report number 1 in [23] (reproductive toxicity section); [26] = Weight of evidence study number 2 in [23] (reproductive toxicity section); [27] = Weight of evidence study number 3 in [23] (reproductive toxicity section).

**Table 15:** Estimation of the systemic DNEL for lead based on animal data. Critical values were taken from the referenced studies summarised in Table 14. Oral absorption rate was taken from the REACH registration dossier for lead [23]. Uncertainty factors and physiological factors were set according to the ECHA procedures for deriving DNELs for threshold endpoints [9] (Table 1 and Table 2). An extra uncertainty factor of 3 was considered for covering uncertainties of extrapolation from oral to inhalation route. N = NOAEL. L = LOAEL. The lowest DNEL is shaded.

	[Study 25] ^1^		[Study 26] ^1^	[Study 27] ^1^
	N	L	L	L
Critical value (mg/kg bw/day)	22.5	45	450	270
Oral absorption rate	0.23	0.23	0.23	0.23
Transformation mg into µg	10^3^	10^3^	10^3^	10^3^
*Uncertainty factors:*				
Interspecies allometric	4	4	7	4
Interspecies remaining	2.5	2.5	2.5	2.5
Intraspecies	10	10	10	10
LOAEL to NOAEL	1	3	3	3
Duration adjustment	1	1	1	1
Additional factor for extrapolation oral-inhalation	3	3	3	3
**Systemic DNEL (µg/kg bw/day)**	17.3	11.5	6.57	69

^1^ See Table 14 for identification of studies.

**Table 16:** Systemic exposure assessment for the chemicals classified as toxic for fertility and sexual function in reference cigarette 1R6F. The mass of each substance was taken from Table 3. A standard adult human weight of 70 kg and a standard consumption of 20 cigarettes/day were assumed.

	Acrylamide	Benzo[a]pyrene	Cadmium	Ethylene Oxide	Lead
Amount/cigarette	4.49 µg	13.8 ng	88.8 ng	17.3 µg	2.81 ng
Cigarettes/day	20	20	20	20	20
Pulmonary absorption (%)	87	56	10	100	25
Weight (kg)	70	70	70	70	70
Systemic exposure	1.1 µg/kg bw/day	2.2 ng/kg bw/day	2.5 ng/kg bw/day	4.9 µg/kg bw/day	0.2 ng/kg bw/day

**Table 17:** Risk characterisation derived from exposure to the chemicals classified as toxic for fertility and sexual function in the reference cigarette 1R6F. The systemic DNELs were taken from Table 5, Table 8, Table 11, Table 13 and Table 15. The systemic exposure was taken from Table 16. The risk was estimated as the exposure/DNEL ratio.

	**Systemic Exposure**	**Systemic DNEL**	**Risk Ratio**
Acrylamide	1.1 µg/kg bw/day	4.7 µg/kg bw/day	0.23
Benzo[a]pyrene	2.2 ng/kg bw/day	36 ng/kg bw/day	0.06
Cadmium	2.5 ng/kg bw/day	14 ng/kg bw/day	0.18
Ethylene oxide	4.9 µg/kg bw/day	370 µg/kg bw/day	0.01
Lead	0.2 ng/kg bw/day	11.5 µg/kg bw/day	0.00002
Mixture			0.48

## Data Availability

Data are contained within the article.

## Supplementary Materials

The following supporting information can be downloaded at: https://www.mdpi.com/article/10.3390/toxics13040234/s1; Supplementary Table S1. Reproductive toxicity data of components of the mixture with a non-harmonized classification on reproductive toxicity endpoint.

## Abbreviations

The following abbreviations are used in this manuscript:

95%CI = 95% confidence interval; ASMR = American Society for Reproductive Medicine; ATSDR = Agency for Toxic Substances and Disease Registry; CTRP = Center for Tobacco Reference Products; DNEL = Derived no-effect level; ECHA = European Chemical Agency; ISO = International Organization for Standardization; LOAEL = Lowest observed adverse effect level; MOS = Margin of safety; NOAEL = No observed adverse effect level; NTP = National Toxicology Program; NTP-CERHR = National Toxicology Program Center for the Evaluation of Risks to Human Reproduction; OR = Odds ratio OR; UFs = Uncertainty factors; PAHs = Polycyclic Aromatic Hydrocarbons; USEPA = United States Environmental Protection Agency; Who = World Health Organization.
