# Supplementary material for: Risk Assessment of Impairment of Fertility Due to Exposure to Tobacco Constituents Classified as Reprotoxicants
Source: Toxics. 2025 Mar 23;13(4):234. doi: 10.3390/toxics13040234 (PMC12031035; doi:10.3390/toxics13040234)
Supplement: Supplementary file 1 [file toxics-13-00234-s001.zip › toxics-3529375-supplementary.pdf]

toxics

MDPI

Article

Risk Assessment of Impairment of Fertility Due to Exposure to Tobacco Constituents Classified as Reprotoxicants

Carmen Estevan[2], Gabriela A. Báez-Barroso[1,3], Eugenio Vilanova[1†] and Miguel A. Sogorb[1,*]

[1] Instituto de Bioingeniería, Universidad Miguel Hernández de Elche, Avenida de la Universidad s/n, 03202-Elche (Spain) alejandra.baez.barroso@gmail.com (GABB);
[2] Departamento de Biología Aplicada, Universidad Miguel Hernández de Elche, Avenida de la Universidad s/n, 03202-Elche (Spain) cestevan@umh.es
3 Current address: Instituto de Investigación Biomédica de A Coruña (INIBIC) Xubias de Arriba, 84, 15006 A Coruña (Spain) alejandra.baez.barroso@gmail.com
* Correspondence: msogorb@umh.es
† Deceased

 https://doi.org/10.3390/toxics13040234

Supplementary Table S1. Reproductive toxicity data of components of the mixture with a non-harmonised classification on reproductive toxicity endpoint. Unless, indicated otherwise, the information was taken from the key study on Toxicity to Reproduction included in the REACH registration dossier database.

Substance	CAS number	NOAEL for reproductive toxicity	LOAEL for reproductive toxicity	NOAEL for parental toxicity
1,3-butadiene	106-99-0	> 6000 ppm	n/a	3000 ppm
1-naphthylamine	134-32-7	Information not available		
2-naphthylamine	91-59-8	Information not available		
3-aminobiphenyl	2243-47-2	Information not available		
4-aminobiphenyl	92-67-1	Information not available		
Acetaldehyde	75-07-0	Study on teratogenic effects. No observed maternal nor developmental toxicity effects at doses up to 400 mg/kg bw		
Acetamide	60-35-5	Information not available		
Acetone	67-64-1	> 10000 mg/L drinking water in 4w study	n/a	> 10000 mg/L drinking water in 4w study
Acrolein	107-02-8	3 mg/kg bw/d	7.2 mg/kg bw/d	1 mg/kg bw/d
Acrylonitrile	107-13-1	> 90 ppm	n/a	15 ppm
Ammonia	7664-41-7	1500 mg/kg bw/d	> 1500 mg/kg bw/d	250 mg/kg bw/d
Benzene	71-43-2	300 ppm	> 300 ppm	300 ppm
Benzo[a]anthracene	56-55-3	Information not available		
Dibenz[a,h]anthracene	53-70-3	Information not available		
Resorcinol	108-46-3	> 3000 mg/L	n/a	> 3000 mg/L
Chromium	7440-47-3	Information not available		
Nickel	7440-02-0	Information not available		
Selenium	7782-49-2	2.5 ppm	7.5 ppm	2.5 ppm
Arsenic	7440-38-2	> 8 mg As_2O_3/m^3		> 8 mg As_2O^3/m^3
Pyrocatechol	120-80-9	> 160 mg/kg bw/d	n/a	30 mg/kg bw/d
Carbon monoxide	630-08-0	Study on teratogenicity, where the embryotoxic NOAEC was considered to be 65ppm. The maternal NOAEC was considered to be 500ppm (highest dose tested).		
Crotonaldehyde	4170-30-3	> 10 mg/kg bw / d	n/a	> 10 mg/kg bw / d
Formaldehyde	50-00-0	< 10 μg/L	Information not available	
Hydrogen cyanide	74-90-8	Information not available		
Hydroquinone	123-31-9	150 mg/kg bw / d		150 mg/kg bw / d
Isoprene	78-79-5	> 6000 ppm (in supporting study)	n/a	300 ppm (in supporting study)
m-Cresol	108-39-4	> 450 mg/kg bw / d		30 mg/kg bw / d
Mercury	7439-97-6	Information not available		
Methyl ethyl ketone	78-93-3	> 2000 ppm	n/a	1000 ppm
N-nitrosoanabasine	37620-20-5	Information not available		

N-nitrosoanatabine	887407-16-1	Information not available		
Nicotine	54-11-5	> 20 µg/L	n/a	> 20 µg/L
1-Butanone, 4-(methyl-nitrosoamino)-1-(3-pyridinyl)-	64091-91-4	Information not available		
N-nitrosonornicotine	80508-23-2	Information not available		
Nitrogen monoxide	10102-43-9	Information not available		
NOx	-	Information not available for NO_2		
o-Cresol	95-48-7	> 450 mg/kg bw/d	n/a	30 mg/kg bw/d
o-Toluidine	95-53-4	Information not available		
p-Cresol	106-44-5	> 450 mg/kg bw/d	n/a	30 mg/kg bw/d
Phenol	108-95-2	> 5000 mg/L	n/a	1000 mg/L
Propionaldehyde	123-38-6	> 1500 ppm	n/a	750 ppm
Propylene oxide	75-56-9	> 300 ppm	n/a	100 ppm
Pyrene	129-00-0	Information not available		
Pyridine	110-86-1	25 mg/kg bw/d	50 mg/kg bw/d	< 12 mg/kg bw/d
Quinoline	91-22-5	Information not available		
Styrene	100-42-5	> 250 ppm	n/a	125 ppm
Toluene	108-88-3	600 ppm	2000 ppm	600 ppm
Vinyl Chloride	75-01-4	> 1100 ppm (in supporting study)	n/a	10 ppm (in supporting study)
